# To be or not to be a case of heparin resistance

**DOI:** 10.1080/20009666.2018.1466599

**Published:** 2018-06-12

**Authors:** Jibran Durrani, Faizan Malik, Naveed Ali, Syed Imran Mustafa Jafri

**Affiliations:** Temple University Hospital affiliate, Philadelphia, PA, USA

**Keywords:** Activated partial thromboplastin time (aPTT), anti-thrombin III (ATIII), activated coagulation time (ACT), unfractionated heparin(UFH), low molecular weight heparin(LMWH), anti-factor 10a (AF 10a), pulmonary embolism (PE), disseminated intravascular coagulation (DIC)

## Abstract

Heparin resistance can be defined as high doses of unfractionated heparin (UFH), greater than 35,000 IU/day, required to raise the activated partial thromboplastin time (aPTT) and activated coagulation time (ACT) to within therapeutically desired ranges or the impossibility of doing so. The most common pathology responsible is the deficiency of anti-thrombin III (ATIII) deficiency. Other clinically relevant conditions that can present with heparin resistance are congenital deficiencies; use of high doses of heparin during extracorporeal circulation, use of asparaginase therapy and disseminated intravascular coagulation (DIC). Most of these conditions effect the ATIII levels. Patients are typically identified in an acute phase, when determination of the cause of resistance is challenging. We present a case where a patient presented with suspected heparin resistance in an acute phase of sickness, where timely intervention was able to prevent a potentially fatal situation.

**Abbreviations**: Neuroendocrine tumors (NETs), World health Organization (WHO), Radiation therapy (RT)

## 1. Introduction

The use of heparin over the past few decades has proven to be a life-saving treatment for many. It has made battling hypercoaguble states convenient and has helped in the form of a prophylaxis medication for clot prevention. The primary effect of heparin is through its interaction with anti-thrombin III (ATIII) [1]. The anticoagulant effect of ATIII is potentiated by a 1000-fold after its transformation when it is bound to heparin molecule [2]. This inactivates thrombin, thus preventing the conversion of fibrin to fibrinogen, and also inactivates activated coagulation factors IX, X, XI and XII. Heparin resistance becomes a concern when greater than 35,000 IUs/day are required to get a subtherapeutic or negligible response. In this situation, the question of true resistance versus a pseudo-resistance becomes relevant to be addressed. The most cost-effective and widely used tests to check for therapeutic levels are activated partial thromboplastin time (aPTT) and activated coagulation time (ACT) [3].

Heparin resistance can result from increased heparin-binding protein levels (acute phase reactants), low ATIII levels (most common cause), increased heparin clearance levels (e.g. due to splenomegaly in liver disease), high factor VIII levels and factitious resistance such as when heparin is not connected to the intravenous line. Table 1 outlines some predictors of heparin resistance [4].

**Table 1.** Predictors of heparin resistance.

(1) AT activity less than or equal to 60%
(2) Platelets greater than 300,000
(3) Age 65 or above
(4) Increased factor VIII and fibrinogen levels

ATIII deficiency is the most common cause of resistance as discussed earlier, it is further subdivided into two types, type I deficiency and type II defects as shown in Table 2.

Acquired ATIII deficiency can be secondary to several pathological conditions resulting in reduced blood levels. Most of the associated conditions cause multiple abnormalities of the coagulation cascade and alter levels of both pro and anti-coagulants [9–11]. Table 3 outlines some of the common causes of acquired ATIII deficiency.

**Table 2.** Phenotypical types of congenital anti-thrombin III deficiencies.

Type I deficiency	Type II defects
● Reduced levels of AT ● Reduced synthesis and or stability secondary to the gene mutations (small deletions or insertions, large deletion or a single base substitution) [5–7,9–11] ● Homozygous cases not reported, Seen to be lethal in mice [8].	● Functionally defective AT ● Mutations leading to reduced activity. ● Three subtypes

**Table 3.** Acquired anti-thrombin III deficiencies.

Causes of Acquired anti-thrombin III deficiencies
Disseminated intravascular coagulation (DIC) Acute thrombosis Liver disease (primarily cirrhosis) Nephrotic syndrome Extracorporeal membrane oxygenation (ECMO) Hemodialysis Surgery and trauma (major surgery associated with nadir of ATIII approx. postoperative day 3 with rise back to normal around day 5) [15,16] Asparaginase therapy, e.g. treatment of ALL patients [18]. Oral contraceptives and estrogen use leading to modest decrease in levels [12,13] Pregnancy. Minimal decrease in levels in normal pregnancy with more significant decrease in levels with pregnancy-induced hypertension, preeclampsia or eclampsia [12,14,17].

Use of heparin can also lower ATIII levels by as much as 30 percent, thought to be secondary to increased clearance of ATIII. This, however, does not increase the risk of thrombosis but can result in reporting of falsely low ATIII levels during the treatment phase with heparin. It is preferred that evaluation and work up for thrombophilia therefore should not be done while undergoing therapy. The two major consequences of low ATIII levels are increased thrombotic risk and insensitivity to heparin.

It has also been observed that surgery and trauma can lead to a modest decrease in the levels of ATIII [15,16]. Major surgery has been associated with a fall in ATIII levels with the levels reaching a nadir around post-operative day 3 followed by rise back to normal around day 5. This decrease in the levels combined with the rise in the acute reactants in the blood can also be a risk factor for thrombus formation and acquired heparin resistance. Also of consideration is the increased clearance of ATIII secondary to use of heparin itself. Though this condition does not increase the thrombotic risk but can result in lower levels than normal, potentially complicating the evaluation of coagulopathy or a hypercoagulable state.

Apparent heparin resistance can be seen in cases of raised factor VIII levels [17]. This is because of the falsely low reported levels of aPTT secondary to raised factor VIII levels. This presents as a peculiar dilemma where the anticoagulant effect of heparin is being exerted but the monitoring is not appropriate. It may be more appropriate to check for plasma concentration of heparin instead, especially if there are concerns of supra-therapeutic levels of heparin and bleeding.

Other causes of low ATIII include the use of asparaganse therapy, especially in acute lymphocytic leukemia (ALL) where it can be a life-saving medication [18]. However, it has been associated with an increased risk of thromboembolic phenomenon. The risk of bleeding might also increase because of the thrombocytopenia. It is unclear if this is a dose dependent risk and or if the presence of central lines contributes to it but usually is associated with decrease in the levels of ATIII levels. Treatment is usually the same as treating for low ATIII levels with replacement therapy.

We present a case where timely intervention was able to prevent complications in a complex medical situation, where the diagnosis of resistance versus pseudo-resistance could have meant the difference between life and death.

## 2. Case description

A 30-year-old African-American male patient presenting to the hospital for trauma injuries to his abdomen. He had sustained a gunshot wound, which necessitated an exploratory laparotomy along with a small bowel resection. The patient had to have an ileocecostomy done. He initially improved but on the third day post operatively he became very short of breath and hypoxic. High flow cannula was initiated and a work up for the possible etiologies of hypoxemia was undertaken. Computed tomographic (CT) scan of the chest with intravenous contrast was performed and was notable for a pulmonary embolism (PE) in third and fourth order segmental branches of the right upper lobe pulmonary artery. Heparin infusion was started for treatment.

The usual protocol with aPTT monitoring, for the dose appropriation of heparin infusion, was initiated. During the following period it was found to be difficult to achieve a therapeutic range for anticoagulation based on the aPTT. During this time he had only been therapeutic once following the transfusion of one unit of fresh frozen plasma. The heparin was titrated up with increasing dose of heparin. However, suspicions for heparin infusion became a concern when the dose required approached 50,000 IU/24 h without aPTT being therapeutic. Haematology was consulted for evaluation and guidance.

At the time of evaluation patient was in no acute distress without any significant physical findings except for tachycardia and abdominal distention with midline scar closed with mesh. The former was likely explained with the PE the latter was secondary to the surgery. The trend for aPTT was noted to be in a sinusoidal pattern ranging between 51 and 27. Since the patient had multiple factors because of the acute situation, it was decided to check for factor 10 levels. Also factor VIII levels were checked. Because of the acute trauma to the abdomen it was suspected the factor VIII might be abnormal. His factor VIII levels were 400% (normal range of 50–200%), ATIII 64% (normal range 75–135%), and anti-factor 10a 0.1 IU/ml (normal therapeutic range goal for P.E 0.3–0.7 IU/ml).

Analysis of the factor levels showed that though the levels of the factor VIII were high, which can contribute to apparently low aPTT, the patient was in fact subtherapeutic, even with 50,000 IU/24 h of heparin. This was supported by lab value for the AF 10a, which was subtherapeutic for the treatment of PE. It was also established that levels of aPTT were not the ideal method of monitoring for heparin dose appropriation. Rather the monitoring of the AF 10a was a better indicator of the dose requirements for heparin. In his situation, the intervention was simple and required increasing the dose of heparin and monitoring AF 10a to the therapeutic range of 0.3–0.7 IU/ml. His dose was further increased and therapeutic levels achieved soon after.

## 3. Discussion

Identification of heparin resistance can present a challenge for professionals, especially since the use is usually in an acute situation. Resistance is to be considered a possibility with the heparin requirement of the infusion exceeds 35,000 IU/day. The causes of heparin resistance need to be considered in each individual independently. Most commonly the appropriation of therapy is based on the aPTT. However, the measured value can be misleading in situations that result in elevated factor VIII level and or with the use of heparin itself. In cases of suspected elevation of factor VIII levels, it may be more appropriate to check for plasma concentration of heparin with the AF 10a levels.

The most common cause of heparin resistance is AT deficiency. The condition can be congenital and or acquired. Testing for the deficiency during the acute phase of illness while patient is on heparin infusion can be misleading. Usually the norm is to test for the AT levels several weeks after the patient has recovered from the acute illness and is not on any form of anticoagulation therapy. This allows for the normalisation of the acute phase reactants that might inadvertently affect the AT levels. The differentiation of the type of the AT deficiency may be a little more challenging. Congenital disease usually presents earlier in life compared to an acquired condition. In a cohort of study the mean age of the first thromboembolic event was noted to be 14 years [19]. On the contrary acquired disease usually manifests at an older age and with an associated condition, such as surgery or nephrotic syndrome. Prevalence of hereditary form is in the range of .02–0.2 percent and prevalence in individuals with venous thromboembolism is approximated to be between 1 and 7 percent. Three forms of therapeutic treatments are available for patients. In situations where the AT concentrates are not available the use of fresh frozen plasma (FFP) may have to suffice. The two forms of AT concentrate available are recombinant and plasma derived AT concentrate. The recombinant form is administered as initial intravenous dose followed by a continuous maintenance infusion. Dosing is based on AT activity monitoring. Whereas, plasma-derived AT concentrate is given as an initial intravenous dose over 10–20 min, followed by AT activity levels in 2 h, and subsequent dosing based on levels. The advantage of using the plasma-derived form is its 24 hourly dosing compared to the continuous infusion, which results from its longer half-life of 43–77 h compared to 10 h [20,21]. However the disadvantage is the risk of blood borne diseases. Normal range for AT is considered to be 80–120%, treatment is beneficial when levels run less than 40–60%. If aPTT is low and heparin resistance is suspected it is recommended to check for AT-heparin cofactor assay. Further, AT-heparin cofactor assay is preferred with factor Xa over testing with IIa.

In cases where heparin resistance is a concern AT-heparin cofactor assay provide a more specific method of testing for heparin dosage approximation and the possibility of heparin resistance. This takes into account low aPTT levels that can be the result of other confounding factors [17]. In such situations the presence of acute phase reactants, rise of factor VIII secondary to trauma or surgery and the use of heparin leading to increased clearance, makes the interpretation of laboratory data difficult. It was the combination of similar confounding factors that made the health care professionals suspect heparin resistance in the mentioned case.
